# Accommodating the cost of growth and swimming in fish—the applicability of exercise-induced growth to juvenile hapuku (*Polyprion oxygeneios*)

**DOI:** 10.3389/fphys.2014.00448

**Published:** 2014-12-01

**Authors:** Javed R. Khan, Caroline Trembath, Steve Pether, Michael Bruce, Seumas P. Walker, Neill A. Herbert

**Affiliations:** ^1^Leigh Marine Laboratory, Institute of Marine Science, The University of AucklandAuckland, New Zealand; ^2^Bay of Plenty PolytechnicTauranga, New Zealand; ^3^National Institute of Water and Atmospheric Research, Bream Bay Aquaculture ParkRuakaka, New Zealand

**Keywords:** exercise training, swim-flume respirometry, aerobic metabolic scope, optimal swim speed, cost of transport

## Abstract

Induced-swimming can improve the growth and feed conversion efficiency of finfish aquaculture species, such as salmonids and *Seriola* sp., but some species, such as Atlantic cod, show no or a negative productivity response to exercise. As a possible explanation for these species-specific differences, a recent hypothesis proposed that the applicability of exercise training, as well as the exercise regime for optimal growth gain (ER_opt growth_), was dependent upon the size of available aerobic metabolic scope (AMS). This study aimed to test this hypothesis by measuring the growth and swimming metabolism of hapuku, *Polyprion oxygeneios*, to different exercise regimes and then reconciling the metabolic costs of swimming and specific dynamic action (SDA) against AMS. Two 8-week growth trials were conducted with ERs of 0.0, 0.25, 0.5, 0.75, 1, and 1.5 body lengths per second (BL s^−1^). Fish in the first trial showed a modest 4.8% increase in SGR over static controls in the region 0.5–0.75 BL s^−1^ whereas the fish in trial 2 showed no significant effect of ER on growth performance. Reconciling the SDA of hapuku with the metabolic costs of swimming showed that hapuku AMS is sufficient to support growth and swimming at all ERs. The current study therefore suggests that exercise-induced growth is independent of AMS and is driven by other factors.

## Introduction

There is ample evidence in the literature showing that induced swimming, or exercise training, can improve the growth and feed conversion efficiency of many species of farmed fish (Davison, 1989; Palstra and Planas, 2011; Davison and Herbert, 2013). Most of this evidence has been accumulated in the salmonid groups *Oncorhynchus* (Houlihan and Laurent, 1987; Aslop and Wood, 1997; Hernández et al., 2002), *Salmo* (Davison and Goldspink, 1977; Totland et al., 1987; Boesgaard et al., 1993) and *Salvelinus* (Leon, 1986; Christiansen et al., 1989; Christiansen and Jobling, 1990) but there are examples of exercise-induced growth from other groups, with species such as the striped bass *Morone saxatilis* (Young and Cech, 1993) and the yellowtail kingfish *Seriola lalandi* (Brown et al., 2011). The global aquaculture industry is expanding rapidly and the potential for continuous exercise to accelerate the growth of fish has direct application due to the potential for fast biomass gain, improved flesh quality and the flexibility of production it can provide. However, exercise-induced growth is often perceived as a paradoxical concept as it seems illogical that fish can expend considerable energy on exercise whilst also committing to the extra expense of accelerated growth. This view is reinforced by a number of studies showing that exercise has either nil, or only negative effects on the growth of fish such as the Atlantic cod *Gadus morhua* (Bjørnevik et al., 2003) and Chinook salmon *Oncorhynchus tshawytscha* (Kiessling et al., 1994). Therefore, to stand any chance of exploiting the economic gains of exercise-induced growth in aquaculture, an in-depth understanding of how fish balance the metabolic costs of growth and exercise needs to be ascertained, particularly in the case of information-poor species that are new to farming.

There has been a rekindled interest in the mechanisms and applicability of exercise-induced growth in recent years (Palstra and Planas, 2011) and new efforts have been made to predict the levels of exercise required for the best rate of growth in novel species using readily accessible measures of behavior and physiology (Davison and Herbert, 2013; Herbert, 2013). In particular, the aerobic metabolic scope (AMS) of fish and the speed where the energetic cost of transport (COT) is at its lowest, termed the optimal swimming speed (*U*_opt_), appears to explain a significant proportion of the variation between different fish that show exercise-induced growth (Davison and Herbert, 2013). AMS is the difference between maintenance and maximal metabolic rates, and thus represents a physiological framework, within which non-maintenance physiological work operates (Jobling, 1994; Clark et al., 2013). In light of this belief, it has been suggested that a larger AMS better accommodates the energetic costs of swimming in addition to other processes, such as protein synthesis associated with growth and feeding (von Herbing and White, 2002; Davison and Herbert, 2013). There are few experimental studies on this topic but the work of Owen (2001) on the European eel *Anguilla anguilla* appears to support this assertion. Indeed, where AMS was insufficient, or the costs of feeding (specific dynamic action, SDA) were deemed excessive, the swimming speed of eels were reduced to accommodate SDA as a form of energetic prioritization (Owen, 2001). On the basis of these observations, Davison and Herbert (2013) examined how the required exercise regime for optimal growth acceleration (termed ER_opt growth_, in units of body lengths s^−1^, BL s^−1^) co-varied with AMS in a variety of well-studied species. As a positive but non-linear correlation was found between ER_opt growth_ and AMS, Herbert (2013) proposed that AMS might have value in predicting the ER_opt growth_ of novel fish species in which the effects of exercise have yet to be investigated. Of particular relevance to those species that show both a positive growth response to exercise and a direct relationship between *U*_opt_ and ER_opt growth_ is the suggestion that that *U*_opt_ speeds are preferentially selected by some migratory fish across extended periods (Hinch and Rand, 2000; Tudorache et al., 2011). This would imply that, when swimming is required, a minimization of swimming costs per unit distance may allow for a greater proportion of available AMS to be allocated to somatic growth. Therefore, to summarize this collective background, fish with a sufficiently high AMS are expected to have the capacity to swim and grow fast at the same time (Davison and Herbert, 2013; Herbert, 2013) and, in this scenario, *U*_opt_ is also believed to predict the best flow regime (speed) for growth (Davison and Herbert, 2013).

In an attempt to test and validate the proposed models of Davison and Herbert (2013) and Herbert (2013), the exercise-induced growth performance of juvenile hapuku, *Polyprion oxygeneios*, a novel farmed finfish species from New Zealand, was quantified and compared against experimentally derived measures of AMS and *U*_opt_. Specifically, if the AMS-ER_opt growth_ model of Davison and Herbert (2013) is applicable to a wider range of species, then the predicted AMS value of hapuku at 17°C (300 mg O_2_ kg^−1^ h^−1^ at 17°C, based on the data of Khan et al., 2014) is hypothesized to provide the metabolic capacity for optimal exercise-induced growth in the vicinity of ~0.4–0.5 BL s^−1^ (Herbert, 2013). If exercise-induced growth is indeed observed in hapuku, *U*_opt_ and ER_opt growth_ should also be relatively well matched (Davison and Herbert, 2013). As a further step in this validation and testing process, the metabolic costs of swimming at different speeds and the recently measured cost of SDA (Khan et al., in press), which is largely comprised of post-absorptive protein synthesis and growth (Secor, 2009; Seth et al., 2010) was also reconciled against the available AMS. This allowed for the experimental resolution of whether hapuku have metabolic capacity to accommodate swimming and the physiological costs associated with growth.

## Materials and methods

### Specimens, tagging, and growth trials

Two full- and half-sibling groups of ~120 juvenile hapuku (*P. oxygeneios*, ~8 months post-hatch, 240 in total) were used for growth trials at the NIWA Bream Bay Aquaculture Facility in Ruakaka, Northland, New Zealand. “Trial 1” fish (128.8 g ± 3.1 g) were hatched 12 weeks prior to “trial 2” fish (172.4 ± 4.5 g) and were also smaller at the start of the growth trials as they were 4 weeks younger at the point when they entered the experimental tanks. Both groups of fish were held at 17°C in larger 4 m^3^ tanks prior to the start of both trials. To track the growth and performance attributes of individuals, all fish were tagged intraperitoneally with a 5 mm passive integrated transponder (PIT) under anesthesia (0.01 mL L^−1^ Aqui-S® followed by 0.3 mL L^−1^ 2-phenoxy-ethanol, standard facility practice). Specimens were treated with chloramine-T (0.005 mL L^−1^) to prevent infection post-tagging (added to flowing tank water, standard facility practice). Any individuals that showed signs of infection were treated further with formalin (0.15 mL L^−1^) or euthanized with an excessive dose of Aqui-S® (0.1 mL L^−1^). Thereafter, two sequential and identical growth trials were conducted incorporating six different exercise regimes (ER, corresponding to six in-tank flow speeds of 0.0, 0.25, 0.5, 0.75, 1, and 1.5 body lengths per second, BL s^−1^). Each of the two trials were conducted in six identical 1.6 m^3^ circular tanks (560 mm water depth, 1900 mm diameter). All tanks were housed in a purpose-built building under ambient light conditions (11L: 13D) and supplied with fresh 1 μm filtered and UV-sterilized (ALX2/8, 150 mW s cm^−2^, Davey Water Products, Australia) seawater at 17 ± 0.3°C. A continuous non-directional inflow of water (30 L min^−1^) was present at the side of each tank and all tanks were central draining. Water flow around the tank was negligible in the 0.0 BL s^−1^ (control) tank but the remaining water flow ER treatments (i.e., 0.25, 0.5, 0.75, 1, and 1.5 BL s^−1^) were maintained through the use of external water pumps (Leader® Ecopool 15, Leader Pumps, Italy) plumbed over the side of each tank via a 25 mm PVC intake and outlet. Pump outlets were connected to a spray bar at a water depth level of 100 mm from the surface and the spray bar extended 500 mm into the tank at a perpendicular angle. Water flow through the spray bars (and thus flow speed in the tanks) was controlled through a ball valve plumbed between the pumps and the spray bars. Flow speeds were set in the tanks by measuring water velocity (in m s^−1^) 200 mm from the tank wall at 100 mm depth on the side directly opposite the spray bar with a Höntzsch® HFA anemometer (V 1.5, Höntzsch technologies, Waiblingen, Germany) and making the necessary correction to the flow of water according to the average body length of the fish at regular fortnightly intervals. Each tank had a single projection of PVC pipe (200 mm high, 100 mm diameter) off the floor, approximately half way between the wall and the center. They were entirely submerged, impossible to remove and created a small low-flow area in their wake. Water chemistry was checked regularly and remained at normal levels at all times throughout both trials.

The fish intended for trial 1 were anesthetized (as described above) in their pre-trial holding tank at 17 ± 0.3°C and their initial weight and length were measured. They were then divided randomly and evenly (~20 per tank) into one of the six experimental tanks and allowed to recover for 4 h with no directional flow. Once swimming behavior appeared normal, flow speeds in the tanks were increased slowly toward one of the six exercise training speeds in BL s^−1^ according to the average BL of all fish in each tank. All tanks were fed to satiation twice a day (at ~0800 and 1600 h) for the following 12 days on Skretting Nova FF 5/7 mm pellets (Skretting, Australia, 50.0% protein, 17.0% lipid, digestible energy 18.6 MJ kg^−1^). Any uneaten feed was recovered 15 min after feeding behavior had ceased (low tank densities allowed feeding behavior to be observed accurately by an observer). The weight of recovered feed was corrected for water absorption by a standard saturation factor (determined by soaking a known weight of feed pellets and then re-weighing, equating to 1.6 × dry weight at saturation). After 12 days all specimens were starved for 48 h and their weight and length recorded under anesthesia (as described above). Water speeds in each tank (other than the control) were then increased to match the increased length of the fish, in order to maintain treatments of 0.25, 0.5, 0.75, 1, and 1.5 BL s^−1^. This was followed by another 12-day period on the same feeding regime. This cycle was repeated twice more to give a total of four 12-day feeding periods interspersed with assessments and adjustments to water speeds. All tanks were treated with Chloramine-T (0.005 mL L^−1^) once per day for 3 days after any handling event and at least 3 h before feeding. There was no measureable difference in feeding behavior between days with Chloramine-T treatments and those without. To ensure ER regimes were maintained at a target level, water flow was checked daily at a position on the opposite side to the spray bar, and at regular spacing intervals around the tank weekly. The weight and length of each individual fish were recorded at the end of the trial under anesthesia (anesthetized as described above).

One week after the end of trial 1, trial 2 commenced and fish were treated in exactly the same way as trial 1 but with ER treatments (0.0, 0.25, 0.5, 0.75, 1, and 1.5 BL s^−1^) randomly reassigned to one of the six tanks. The only other exception was a reduction in the number of fish per tank in the second trial (17 fish per tank in trial 2 vs. ~20 per tank in trial 1) and therefore a difference in biomass density between trials (trial 1 = 1.99 ± 0.12 kg m^−3^; trial 2 = 2.03 ± 0.22 kg m^−3^).

Mass specific growth rate (SGR, % body weight day^−1^) was calculated for each individual using the formula:

$$\text{SGR}=(\text{ln}m_{2}-\text{ln}m_{1})/({\text{t}}_{2}-{\text{t}}_{1})\times 100$$

where, *m*_1_ is the initial weight at the start of the growth period t_1_ and *m*_2_ is the final weight at the end of the growth period t_2_.

Feed conversion ratio (FCR), measured as the weight of dry feed intake (corrected for uneaten feed) per unit weight gain for the period, and was calculated for each tank using the following formula:

$$\begin{array}{l} \text{FCR}=\text{weight of dry feed consumed in tank}/\\ \text{ wet weight gained in tank} \end{array}$$

The initial and final condition factor (CF) of fish was also calculated using the formula:

$$\text{CF}=\text{mass}/{\text{length}}^{3}\times 100$$

The relative change in CF (ΔCF) over the course of the growth trials was then calculated as the difference between final and initial CF.

### Respirometry

Swim flume respirometry was performed on fish from three ER treatments (0.0, 0.75, and 1.5 BL s^−1^) to resolve the effect of exercise training on metabolic cost functions. Aside from understanding the potential metabolic effects of long-term exercise, this information was important for reconciling the cost of growth and swimming. All specimens were starved for 48 h prior to respirometry to remove any confounding effects of feeding on metabolic rate (Ross et al., 1992; Thuy et al., 2010). The mass specific rate of oxygen consumption (*M*O_2_, mg O_2_ kg^−1^ h^−1^) was then determined from 24 fish from trial 2 (i.e., 8 fish from the 0.0, 0.75 and 1.5 BL s^−1^ ER groups) over a period of 30 days in the 38.4 L Brett-type swim flume respirometer described by Brown et al. (2011). The change in oxygen saturation in the respirometer was measured continuously using a Firesting® 2-channel oxygen meter (Pyroscience, Germany) connected to an oxy-dipping probe (Pyroscience, Germany) which was sealed into the respirometer in a position anterior to the swimming section. The respirometer was operated through a custom software interface which controlled water flow speed in the swimming section and the cycling of the flush, wait and measure periods (5, 1 and 4 min, respectively, 10 min total). *M*O_2_ and its components were calculated using the same formulae as Brown et al. (2011).

After measuring the weight, length, depth, and width of fish [to compensate for the solid-blocking effect (Steffensen, 1989)], specimens were placed in the sealed swimming section of the respirometer (530 × 130 × 155 mm). This occurred at ~1600 h and provided fish an overnight period of acclimation to the conditions of the respirometer with a low flow of water (0.25 BL s^−1^) and with the system cycling automatically through a repeated series of flush, wait and measure. From 0800 the following day, a critical swimming speed (*U*_crit_) test commenced where the flow speed inside the swimming section was increased by 0.25 BL s^−1^ every 30 min (i.e., after three 10 min flush-wait-measure cycles). This continued until ⅓ of the body was pressed up against the rear of the swimming section or erratic and non-directional burst activity was observed. Fish swimming behavior was monitored at all times with a CCD camera (KT & C 19 mm, Seoul, Korea) attached to an external monitor. After each experiment was complete, background oxygen consumption levels were measured without a fish and confirmed that bacterial respiration was essentially nil and negligible in all runs. All equipment was cleaned thoroughly between experiments with freshwater and a mild hypochlorite solution (0.005 g L^−1^).

For each individual fish, critical swimming speed (*U*_crit_) was calculated using the same formula as Brett (1964), Brown et al. (2011), and Yanase et al. (2012). The 15% quantile method of Chabot and Claireaux (2008) and Franklin et al. (2013) was used to obtain a near-resting value of *M*O_2_ from overnight measures at 0.25 BL s^−1^ in order to remove erroneously-low vales associated with unusually weak oxygen probe signals. Thereafter, three *M*O_2_ values obtained from each of the three flush-wait-measure cycles at each speed were averaged to resolve the relationship between swimming speed and *M*O_2_ (Korsmeyer et al., 2002; Brown et al., 2011) at speeds that were considered to be exclusively aerobic, i.e., up to 2.5 BL s^−1^ (Roche et al., 2013). In order to yield an estimate of standard metabolic rate (*M*O_2standard_) for every individual fish, average *M*O_2_ at all speeds was extrapolated back to 0.0 BL s^−1^ using an exponential regression function as used previously by other authors (Pettersson and Hedenström, 2000; Yanase et al., 2012) as power functions underestimated *M*O_2standard_ values compared to other investigations on the same species (Khan et al., 2014, in press). Using all *M*O_2_ values from the point that fish first entered the respirometer, *M*O_2max_ was calculated using the 99% quantile method of Khan et al. (2014) as this yielded higher, and produced less inter-individual variation, than *M*O_2_ values at *U*_crit_. AMS was calculated by subtracting *M*O_2standard_ from *M*O_2max_ and the gross cost of transport (GCOT, mg O_2_ kg^−1^ BL^−1^) was calculated by dividing *M*O_2_ by their corresponding swimming velocity (BL s^−1^).

### Statistical analyses

The data relating the effect of ER treatments on SGR, ΔCF, FCR and feed per individual (g) from trial 1 and 2 were each initially described with a second-order (non-linear) polynomial regression of the form: *y* = *ax*^2^ + *bx* + *c*. Non-linear polynomial regressions were also used to test the effect of ER on GCOT, as well as being used to calculate *U*_opt_ (Pettersson and Hedenström, 2000). Exponential regressions were used to analyse the effect of ER on the relationship between *M*O_2_ and swimming speed. Due to the presence of non-normal data, the effect of ER on SGR and ΔCF in both trials was tested with a non-parametric Kruskal–Wallis One-Way analysis of variance (ANOVA) test. When this test identified a significant effect of ER, a Dunn's comparison test was then used to locate a specific *post-hoc* difference in SGR or ΔCF from the control 0 BL s^−1^ ER treatment. After ensuring that data was compliant for normality and homoscedasticity, a repeated measures (RM) Two-Way ANOVA was used to test the effect of swim speed on *M*O_2_ (factor 1) as well as the effect of long-term ER on *M*O_2_ (factor 2). The same Two-Way RM ANOVA was also used to test the effect of the same two factors on GCOT. The optimal (i.e., least cost) swimming speed (*U*_opt_) of individual fish was calculated from the non-linear speed-GCOT regression and taken as the speed that yielded a minimum level of GCOT. The effect of long-term ER on *U*_opt_, *M*O_2standard_, *M*O_2max_, AMS, and *U*_crit_ was then tested with individual One-Way ANOVA tests, followed by a Tukey *post-hoc* test for specific pairwise comparisons where appropriate. Significance was accepted at *P* ≤ 0.05 and all data are displayed ± standard error. All statistical analyses were performed using SigmaPlot® version 11.0.

## Results

### Effects of exercise training on juvenile hapuku growth

**Figure 1:**
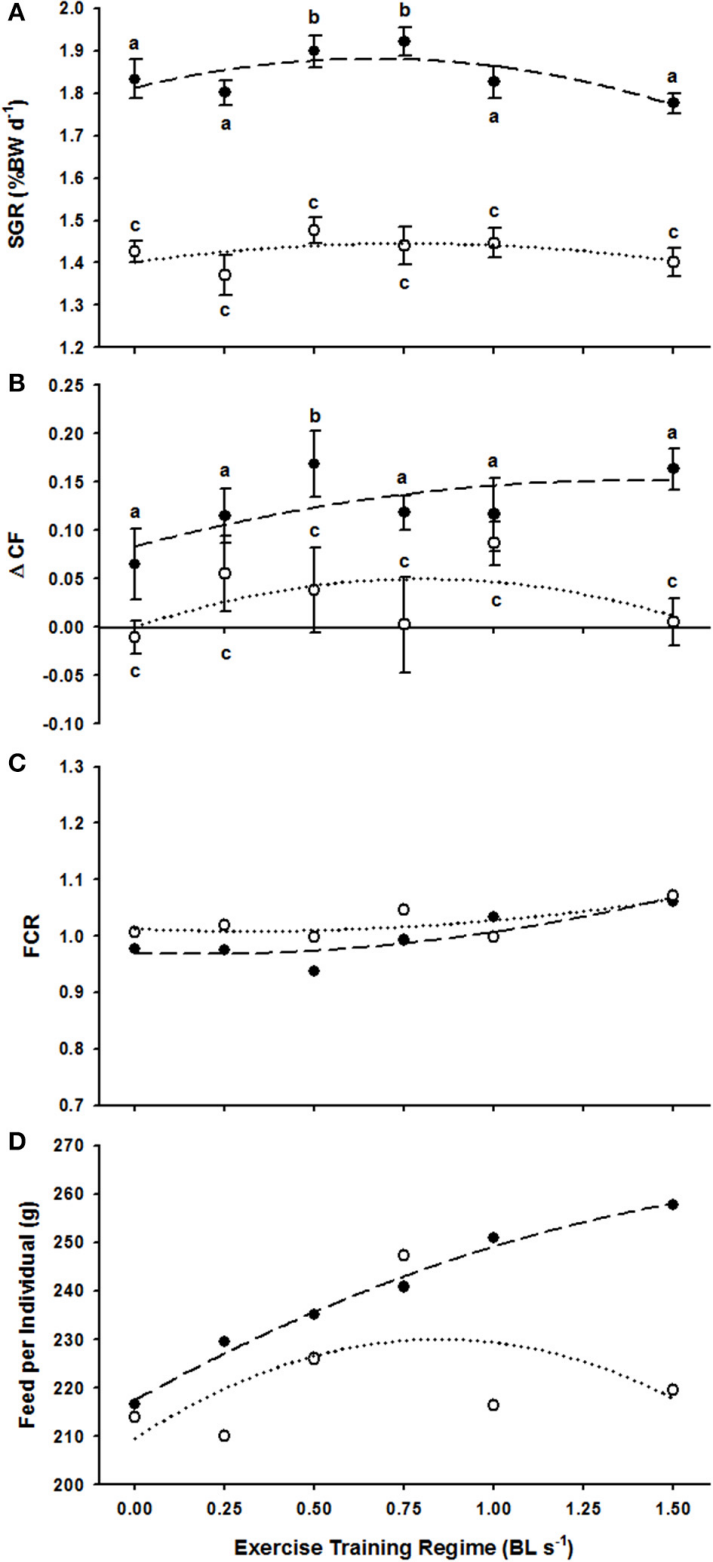
**The effect of exercise regimes (ER, in BL s^−1^) on various production parameters of *P. oxygeneios* in trial 1 (closed circles, broken line) and trial 2 (open circles, dotted line). (A)** Average SGR. Regressions are second order non-linear polynomials described as: *y* = −0.155*x*^2^ + 0.205*x* + 1.813 and *y* = −0.075*x*^2^ + 0.114*x* + 1.402 for the trial 1 and 2, respectively. **(B)** Average ΔCF. Regressions are: *y* = −0.035*x*^2^ + 0.098*x* + 0.083 and *y* = −0.078*x*^2^ + 0.125*x* for trial 1 and 2, respectively. **(C)** Feed conversion ratio, FCR. Regressions are: *y* = 0.058*x*^2^ − 0.02*x* + 0.97 and *y* = 0.0401*x*^2^ − 0.025*x* + 1.013 for trial and 2 fish, respectively. **(D)** Feed intake per individual (g). Regressions are *y* = −9.390*x*^2^ + 41.138*x* + 217.51 and *y* = −28.539*x*^2^ + 48.304*x* + 209.61 for the trial 1 and 2 fish, respectively. Dissimilar letters in each of the plots represent a significant difference between swim speed treatments (*P* < 0.05).

Non-linear regressions did not provide convincing evidence that ER was positively linked with weight-specific growth (SGR) for either trial 1 (*F* = 1.91, *R*^2^ = 0.56, *P* > 0.05) or trial 2 (*F* = 0.56, *R*^2^ = 0.27, *P* > 0.05) (Figure 1A). Kruskal–Wallis tests confirmed that ER did not have any effect on the SGR of fish in Trial 2 (*H* = 5.23, *P* > 0.05) where starting weights were higher (Table 1) but a strong positive effect of ER on the SGR of fish in trial 1, where starting weights were lower, was identified (*H* = 18.93, *P* < 0.01) (Figure 1A and Table 1). Specific *post-hoc* comparisons against the control 0.0 BL s^−1^ treatment revealed that fish were subject to a significant 3.5% increase in SGR at 0.5 BL s^−1^ (*P* < 0.05) and a 4.8% increase in SGR at 0.75 BL s^−1^ (*P* < 0.05) (Figure 1A). No other ER treatment was subject to a change in SGR.

**Table 1:** **The starting weight (g), final weight (g), total feed consumed (g), and number of fish in each of Trial 1 and Trial 2**.

**Tank speed (BL s^−1^)**	**Trial 1**				**Trial 2**			
	**Start weight (g)**	**End weight (g)**	***N***	**Total feed intake (g)**	**Start weight (g)**	**End weight (g)**	***N***	**Total feed intake (g)**
0.0	119.9 ± 3.1	341.4 ± 10.1	20	4331.4	170.2 ± 6.1	382.6 ± 12.1	17	3635.2
0.25	131.4 ± 4.7	366.7 ± 9.9	19	4360.1	170.2 ± 8.0	372.8 ± 5.6	17	3359.3
0.5	128.6 ± 4.6	380.6 ± 12.6	20	4702.2	170.9 ± 5.7	397.2 ± 14.1	17	3841.9
0.75	122.7 ± 4.6	365.2 ± 11.8	19	4576.2	187.5 ± 10.6	423.8 ± 19.7	17	4205.7
1.0	133.0 ± 4.3	375.8 ± 7.1	16	4015.2	171.8 ± 10.1	388.4 ± 19.0	17	3678.9
1.5	138.8 ± 3.1	381.9 ± 7.6	17	4384.3	167.4 ± 6.2	372.3 ± 6.2	17	3732.4

*All values shown ± standard error*.

The regressions detailing the link between ER and ΔCF were non-significant within the scale of responses observed in trial 1 (*F* = 1.33, *R*^2^ = 0.47, *P* > 0.05) and trial 2 (*F* = 0.65, *R*^2^ = 0.3, *P* > 0.05) (Figure 1B). ANOVA tests revealed that ΔCF was positively affected by increasing ER in both trial 1 (*H* = 12.29, *P* < 0.05) and trial 2 (*H* = 13.76, *P* < 0.05). However, specific *post-hoc* comparisons against the 0.0 BL s^−1^ control only revealed a significantly higher ΔCF following long-term swimming at 0.5 BL s^−1^ in trial 1 (Figure 1B). Therefore, in addition to the positive effect on SGR, fish at 0.5 BL s^−1^ had a relatively deeper body shape.

FCR varied little as a function of ER across trial 1 (*F* = 4.98, *R*^2^ = 0.77, *P* > 0.05) and trial 2 (*F* = 1.67, *R*^2^ = 0.53, *P* > 0.05) (Figure 1C). Feed intake per individual (g) was positively related to ER in trial 1 fish (*F* = 115.48, *R*^2^ = 0.98, *P* < 0.05) but showed no relationship with ER in trial 2 (*F* = 0.78, *R*^2^ = 0.34, *P* > 0.05, Figure 1D).

### Effects of exercise training on the swimming performance of juvenile hapuku

**Figure 2:**
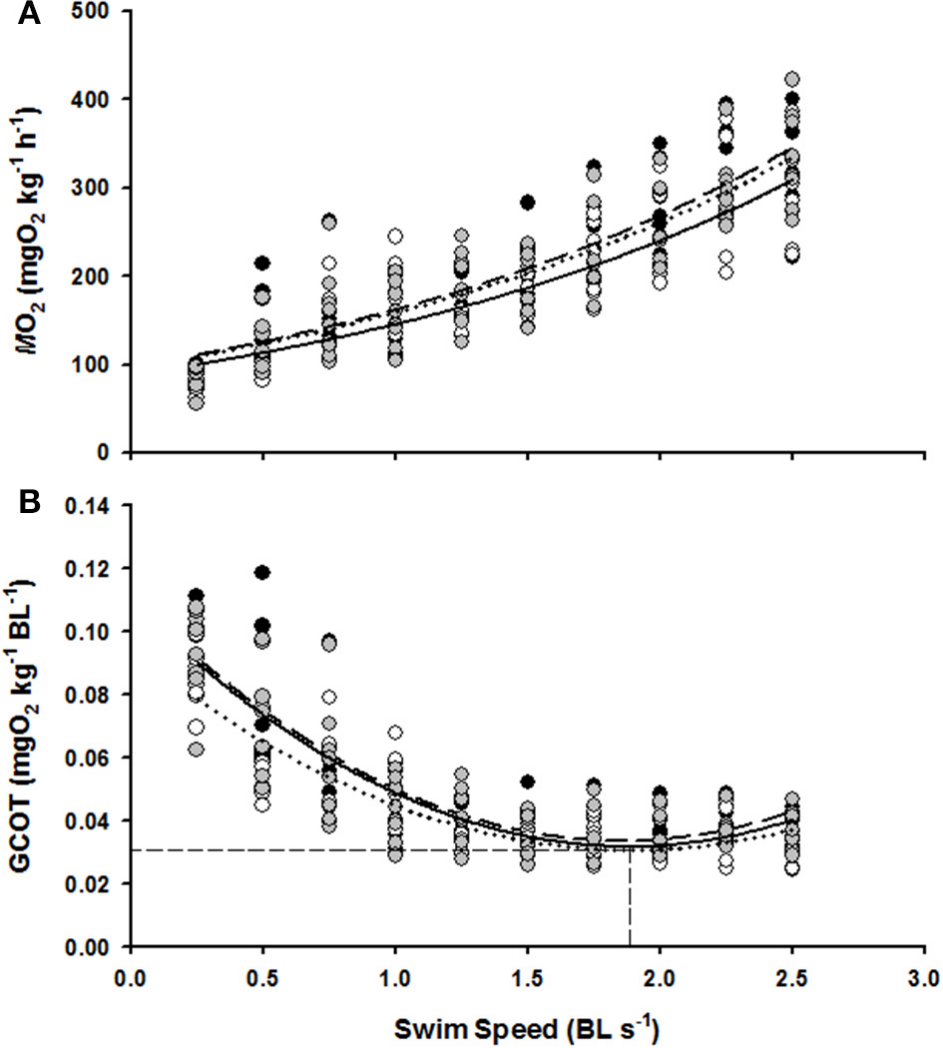
**The effect of swim flume speed (BL s^−1^) on the *M*O_2_ (A) and GCOT (B) of juvenile hapuku raised for 6 weeks at either 0.0 BL s^−1^ (black circles, broken line), 0.75 BL s^−1^ (open circles, dotted line), or 1.5 BL s^−1^ (gray circles, solid line)**. *M*O_2_ regressions are *y* = 90.38*e*^0.5438*x*^, *y* = 79.47*e*^0.5531*x*^, and *y* = 89.26*e*^0.5399*x*^, respectively. GCOT regressions are *y* = 0.023*x*^2^ − 0.084*x* + 0.111, *y* = 0.018*x*^2^ − 0.067*x* + 0.094, and *y* = 0.021*x*^2^ − 0.081*x* + 0.109, respectively. As no significant differences were detected between ER treatments in GCOT (see Results), the horizontal dashed lines refers to the calculated pooled GCOT_min_ (0.03 mg O_2_ kg^−1^ BL^−1^) and the vertical dashed line refers to pooled *U*_opt_ (1.86 BL s^−1^).

*M*O_2_ increased linearly with swimming speed for each of the 0.0, 0.75, and 1.5 BL s^−1^ ER groups (linear regressions with *R*^2^ = 0.78, *R*^2^ = 0.78, *R*^2^ = 0.76, and *P* < 0.05 for the 0.0, 0.75, and 1.5 BL s^−1^ ER groups, respectively, Figure 2A) and a highly significant effect of swimming speed on *M*O_2_ was confirmed from the Two-Way RM ANOVA tests (*F* = 136.11, *P* < 0.01). There was, however, no significant difference in *M*O_2_ between the three ER treatments (*F* = 1.41, *P* > 0.05) and there was no significant interaction between swimming speed and ER on *M*O_2_ (*F* = 0.61, *P* > 0.05).

GCOT showed a significant parabolic relationship with swimming speed for each of the 0.0, 0.75, and 1.5 BL s^−1^ ER groups (*R*^2^ = 0.74, *R*^2^ = 0.73, *R*^2^ = 0.73, and *P* < 0.05 for the 0.0, 0.75, and 1.5 BL s^−1^ ER groups, respectively, Figure 2B) and a highly significant effect of swimming speed on GCOT was once again confirmed with the Two-Way RM tests (*F* = 138.47, *P* < 0.01). However, there was no significant difference in GCOT between the three ER groups (*F* = 0.51, *P* > 0.05) and there was no interactive effect of swimming speed and ER on GCOT (*F* = 0.46, *P* > 0.05). *U*_opt_ estimations were also not significantly different between the three ER treatments (*F* = 1.26, *P* > 0.05) and were essentially identical to the pooled *U*_opt_ estimation of 1.86 BL s^−1^ with a GCOT minima of 0.03 mg O_2_ kg^−1^ BL^−1^.

Long-term exposure to the three ER treatments had no significant effect on *M*O_2standard_ (*F* = 1.17, *P* > 0.05), *M*O_2max_ (*F* = 1.15, *P* > 0.05), AMS (*F* = 0.75, *P* > 0.05), or *U*_crit_ (*F* = 2.63, *P* > 0.05) (Table 2).

**Table 2:** **The average weight (g), standard metabolic rate (*M*O_2standard_), maximum metabolic rate (*M*O_2max_), and aerobic metabolic scope (AMS) of juvenile hapuku (measured as mg O_2_ kg^−1^ h^−1^) as well the critical swimming speed (*U*_crit_, BL s^−1^) of juvenile hapuku raised for 6 weeks at either 0.0, 0.75, or 1.5 BL s^−1^ and measured in a swim-flume respirometer**.

	**Swim speed treatment**		
	**0.0 BL s^−1^**	**0.75 BL s^−1^**	**1.5 BL s^−1^**
Average weight (g)	469.12 ± 5.96	496.38 ± 8.14	481.44 ± 6.77
Standard metabolic rate (*M*O_2standard_, mg O_2_ kg^−1^ h^−1^)	91.33 ± 5.37	80.58 ± 5.46	90.67 ± 5.80
Maximum metabolic rate (*M*O_2max_, mg O_2_ kg^−1^ h^−1^)	324.72 ± 7.44	294.05 ± 5.99	337.90 ± 8.59
Aerobic metabolic scope (AMS, mg O_2_ kg^−1^ h^−1^)	233.39 ± 9.04	213.47 ± 5.49	247.23 ± 8.23
Critical swimming speed (*U*_crit_, BL s^−1^)	2.72 ± 0.13	2.55 ± 0.08	2.94 ± 0.14

*No significant effect of swimming speed treatment was detected in any of the listed variables (P > 0.05)*.

*All values shown ± standard error*.

### Reconciling the cost of swimming and growth

**Figure 3:**
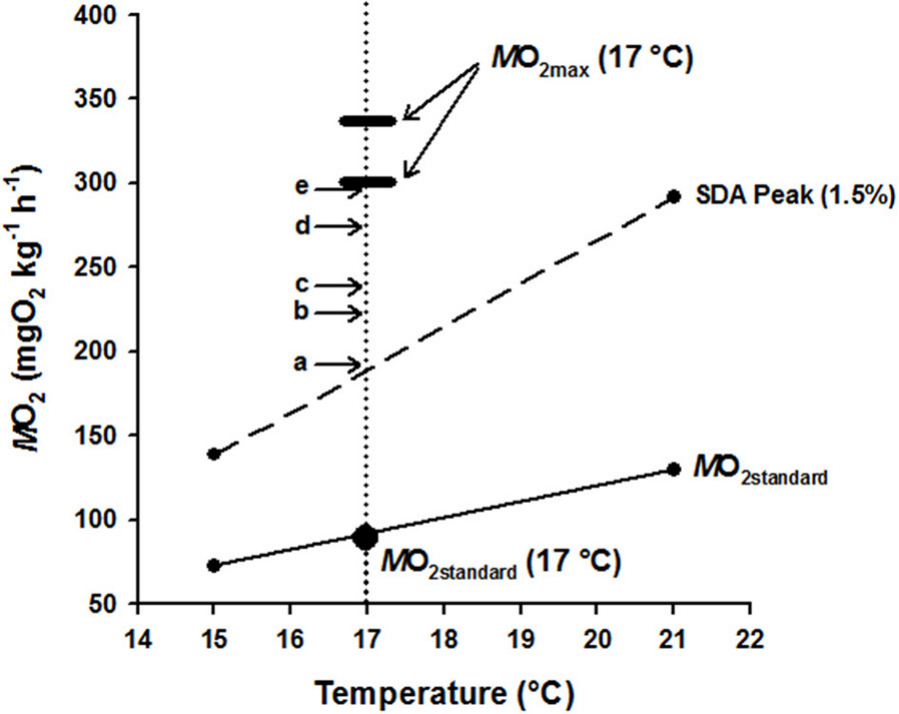
**Graphical summary of hapuku metabolic components as a function of temperature (15–21°C) with particular detail at 17°C, allowing the costs of specific dynamic action (SDA) and swimming to be balanced within the boundaries of aerobic metabolic scope (AMS = *M*O_2max_ - *M*O_2standard_)**. *M*O_2standard_ at and between 15 and 21°C (small filled circles adjoined by the lowest solid line) was taken from Khan et al. (in press) using a static respirometry system whilst *M*O_2standard_ at 17°C (large filled circle) was measured directly within the current study using the swim-flume respirometer. The two small filled circles adjoined by the broken line show the peak in the SDA response of 300–500 g hapuku fed a 1.5% BW d^−1^ ration at and between 15 and 21°C (Khan et al., in press). It is likely that the SDA costs measured at 15 and 21°C can be used to accurately interpolate the feeding costs of fish in the current study at 17°C because the fixed 1.5% BW d^−1^ ration of Khan et al. (in press) closely approximates the 1.3–1.8% BW d^−1^ *ad libitum* ration level of fish from the different ER treatments. The thickened horizontal dash indicates the *M*O_2max_ of 300–500 g hapuku measured in the swim flume at 17°C during the current study. Overlaid above *M*O_2standard_ (solid line) and peak SDA (broken line) are horizontal arrows with letters (a–e) showing the additional measured costs of swimming from the current study at 17°C as follows: (a) 0.25 BL s^−1^, (b) 0.5 BL s^−1^, (c) 0.75 BL s^−1^, (d) 1.0 BL s^−1^, and (e) 1.5 BL s^−1^. The vertical dotted line therefore represents the transect through the accumulated costs of maintenance (*M*O_2standard_), feeding/growth (SDA) and exercise of *P. oxygeneios* at 17°C and shows that the costs of SDA and exercise fall comfortably within the limits of available aerobic metabolic scope at this temperature (see Results and Discussion for more detail).

A summary of hapuku metabolic costs across a temperature range of 15–24°C was amalgamated and graphically represented (Figure 3) for the purpose of reconciling metabolic components against available AMS at 17°C. *M*O_2standard_ values for 15°C and 21°C were measured in a different study in similarly sized fish using a static respirometry system (Khan et al., in press) and the line between these two values intersected 17°C at 91.31 mg O_2_ kg^−1^ h^−1^ which is very similar to the *M*O_2standard_ estimate from the current study (87.53 ± 5.21 mg O_2_ kg^−1^ h^−1^) at 17°C (Table 2). SDA estimates were also measured in the same previous study for fish fed a 1.5% BW d^−1^ ration at both 15 and 21°C (Khan et al., in press). An estimate of SDA at 17°C was then interpolated from the straight line function between these two SDA values (*Q*_10_ = 3.44, *M*O_2_ = 25.45*temp* – 242.95). It was therefore assumed that peak SDA follows a linear relationship between these two temperatures when fed the same-sized ration. (NB. ration size varied 1.3–1.8% BW d^−1^ in the current study so was close to the standard 1.5% BW ration in Khan et al., in press). ER had no significant effect on swimming costs at 17°C (see above) so the *M*O_2_ values from each ER were pooled to calculate an average cost of swimming at 0.25, 0.5, 0.75, 1.0, and 1.5 BL s^−1^. *M*O_2max_ values are shown as the highest and lowest estimates from the current study (i.e., 1.5 BL s^−1^ = 337.90 ± 8.59 mg O_2_ kg^−1^ h^−1^ and 0.75 BL s^−1^ = 294.05 ± 5.99 mg O_2_ kg^−1^ h^−1^, respectively, Table 2).

## Discussion

In order to validate the model of Davison and Herbert (2013), exercise-induced growth in juvenile hapuku would be expected in the range of ~0.4–0.5 BL s^−1^. However, the current does not provide compelling evidence of exercise-induced growth at 17°C (Figure 1) which is a stark contrast to salmonids that reportedly show a ≤40% increase in growth from sustained exercise in the region of 0.75–1.5 BL s^−1^ (Davison and Goldspink, 1977; Houlihan and Laurent, 1987; Jørgensen and Jobling, 1993). Indeed, hapuku with an average starting weight of 130 g in Trial 1 only showed a maximum of a 4.8% increase in growth at 0.5 and 0.75 BL s^−1^, respectively, (Figure 1A) whereas the larger 170 g (starting weight) hapuku showed no sign of exercise-induced growth in Trial 2 (Table 1). Earlier studies on salmonids considered that the ER at which optimal growth is ascertained (i.e., ER_opt growth_) was attributed to their active ecotype, as well as the physiological and behavioral requirements of schooling, migration and river spawning [e.g., position holding in strong water flows (Jobling et al., 1993)]. Hapuku would not be described as highly active so the data is consistent with the view of rgensen (Jobling et al., 1993). However, the recent review on exercise-induced growth in fish by Davison and Herbert (2013) went further to propose that ER_opt growth_ is a function of AMS. Most salmonids, with their active ecotype and high AMS (~350–500 mg O_2_ kg^−1^ h^−1^), show exercise-induced growth at relatively fast swimming speeds (Walker and Emerson, 1978; Houlihan and Laurent, 1987; Jørgensen and Jobling, 1993; Bugeon et al., 2003) and therefore provide data to support the upper end of the Davison and Herbert (2013) model. In contrast, the lower end of the model is based on species such as gadoids that have a small AMS in the region of ~150–200 mg O_2_ kg^−1^ h^−1^ (Hammer, 1994; Karlsen et al., 2006) and show little to no growth response to exercise-training (Bjørnevik et al., 2003; Karlsen et al., 2006). On the basis of these observations, the current study aimed to assess the AMS – ER_opt growth_ model of Davison and Herbert (2013) by testing whether the ~300 mg O_2_ kg^−1^ h^−1^ AMS level of Khan et al. (2014) does indeed lead to an ER_opt growth_ of 0.4–0.5 BL s^−1^. At least for trial 1, the Davison and Herbert (2013) model prediction does appear to provide a reasonable fit. However, the lack of exercise-induced growth in trial 2 is not consistent with the Davison and Herbert (2013) model and the very modest levels of growth acceleration do not validate the model for this novel species.

The AMS values measured from 480 g hapuku at the end of the current study ranged from 213 to 247 mg O_2_ kg^−1^ h^−1^ (Table 2) and are therefore lower than the 300 mg O_2_ kg^−1^ h^−1^ AMS value ascertained for 180 g hapuku at 17°C in the study of Khan et al. (2014). Whilst these larger AMS values were used initially to formulate our hypothesis, the recently established values of AMS are considered more valid because they originate from a size class of fish that corresponds to the current ER_opt growth_ data. However, applying these lowered AMS values to the model of Davison and Herbert (2013) predicts an ER_opt growth_ of between 0.15 and 0.3 BL s^−1^ which does not correspond to the observed ER_opt growth_ range of fish in trial 1, or even the total lack of exercise-induced growth in trial 2 (Figure 1A). These data further suggest that the relationship between AMS and ER_opt growth_ is not validated in this species.

In relation to the second model of Davison and Herbert (2013), the ER_opt growth_ range observed in trial 1 (0.5–0.75 BL s^−1^, Figure 1) does not even vaguely correspond to the measures of *U*_opt_ in the current study (1.86 BL s^−1^, Figure 2B). The *U*_opt_ estimation for juvenile hapuku was unaffected by ER and is considerably higher than one might expect for a species that is less active than Atlantic salmon *Salmo salar*, brown trout *Salmo trutta*, and brook charr *Salvelinus fontinalis* which all have *U*_opt_ values in the range of 0.9–1.1 BL s^−1^ (Beaumont et al., 2000; Deitch et al., 2006; Tudorache et al., 2011). Atlantic cod and gilthead seabream *Sparus aurata* also have unusually high *U*_opt_ estimations [ranging from 1.2 to 1.6 BL s^−1^ in the cod and up to 2.3 BL s^−1^ in the gilthead seabream (Schurmann and Steffensen, 1997; Steinhausen et al., 2010)]. Alternative methods of calculating the minimum COT (i.e., those suggested by Pettersson and Hedenström, 2000) produce a similarly high *U*_opt_ estimate of 1.84 BL s^−1^ for the pooled GCOT data (Figure 2B). It may be that these less active ecotypes do not have an ecologically functional or relevant *U*_opt_ as would be the case for migratory or highly active species (Hinch and Rand, 2000; Tudorache et al., 2011) though this is speculation and requires further investigation.

The hypothesis that AMS places a capacity limitation on exercise-induced growth (Davison and Herbert, 2013) is not supported by the current data for juvenile hapuku. For 480 g hapuku at 17°C, the costs of exercise and SDA [which can be comprised of up to 80% protein synthesis (Coulson and Hernandez, 1979; Brown and Cameron, 1991; Seth et al., 2010; Li et al., 2013)] are easily accommodated within available AMS, even at the highest swimming speed used in the growth trials (1.5 BL s^−1^, Figure 3). It is generally accepted that the energetic costs associated with SDA are largely comprised of post-absorptive protein synthesis and is thought to represent the cost of growth (Whiteley et al., 2001; Grigoriou and Richardson, 2008; Secor, 2009) and, in less active species with low AMS, SDA often consumes a large proportion of AMS potential (Jobling, 1983; Soofiani and Priede, 1985; Jordan and Steffensen, 2007). This has led researchers to propose that an inability to reconcile the metabolic costs of growth and exercise simultaneously would either lead to a reduction in the rate of protein synthesis (as a prioritization of exercise over growth, Davison and Herbert, 2013) or, as predicted for the European eel *Anguilla anguilla* in the study of Owen (2001), a reduction in swimming activity as a prioritization of growth over exercise. Therefore, with an ability to accommodate the costs of exercise and growth simultaneously and with metabolic costs of swimming (Figure 2) and SDA not vastly different to other ecotypes (Fu et al., 2005; Jordan and Steffensen, 2007; Ohlberger et al., 2007; Yanase et al., 2012; Frisk et al., 2013), it is proposed that the weak exercise growth response of hapuku is a species-specific effect and not due to capacity limitation of aerobic metabolism.

The data in Figure 3 provides evidence that AMS does not limit the ability of juvenile hapuku to swim and grow simultaneously but, on a cautionary note, it does not take into the account the extra metabolic costs of spontaneous activity (Boisclair and Tang, 1993; Tang et al., 2000) nor does it necessarily prove that hapuku have the metabolic capacity to grow *faster* whilst swimming. With respect to the latter point, the SDA costs of supplementary fast growth from exercise were not measured within static respirometry chambers (Khan et al., in press) and is therefore still not yet resolved. Interestingly, there is other recent data suggesting that the costs of SDA and exercise can act additively in the darkbarbel catfish *Peltebargus vachelli* (Li et al., 2010) and the sea bass *Dicentrarchus labrax* (Altimiras et al., 2008) to the point where total costs exceed measured *M*O_2max_. This is relevant to the current discussion as it suggests a potential disconnect between AMS and the combined costs of exercise and growth. More importantly, this data opposes the AMS - ER_opt growth_ hypothesis of Davison and Herbert (2013) as exercise and growth could potentially occur simultaneously in catfish and sea bass without their costs being limited by AMS. The presence of additive SDA has not yet been addressed in hapuku and, whilst this species appears suited to *U*_crit_ swimming tests in a swim-flume respirometer, feeding attempts have not yet been successful. To investigate this issue further, it may be necessary to implement a gavage protocol or directly infuse food or amino acids into the gut or bloodstream (Brown and Cameron, 1991; Li et al., 2010).

## Conclusion

The data from the current study is not consistent with the hypothesis of Davison and Herbert (2013) that AMS sets a limit to, and therefore determines, the likelihood of seeing exercise-induced growth in finfish aquaculture species such as hapuku. This was essentially based on the fact that (i) juvenile hapuku showed a modest and inconsistent exercise-induced growth response in a narrow band of swimming speeds (0.5–0.75 BL s^−1^), and (ii) the AMS of these fish appears sufficient to accommodate the physiological costs SDA and swimming simultaneously. It may be that this species is generally not responsive to exercise training but, before that conclusion is reached, future research should possibly strive to examine the response of different-sized hapuku across a greater range of (optimal) temperatures as a means of disentangling the potential role of these factors in exercise-induced growth (e.g., Brown et al., 2011).

### Conflict of interest statement

The authors declare that the research was conducted in the absence of any commercial or financial relationships that could be construed as a potential conflict of interest.
